# Mechanobiology in the Comorbidities of Ehlers Danlos Syndrome

**DOI:** 10.3389/fcell.2022.874840

**Published:** 2022-04-25

**Authors:** Shaina P. Royer, Sangyoon J. Han

**Affiliations:** ^1^ Department of Biomedical Engineering, Michigan Technological University, Houghton, MI, United States; ^2^ Department of Mechanical Engineering, Michigan Technological University, Houghton, MI, United States; ^3^ Health Research Institute, Michigan Technological University, Houghton, MI, United States

**Keywords:** mechanobiology, classical Ehlers-Danlos syndrome, hypermobile Ehlers-Danlos Syndrome, collagen V, extracellular matrix, stiffness, mast cell degranulation

## Abstract

Ehlers-Danlos Syndromes (EDSs) are a group of connective tissue disorders, characterized by skin stretchability, joint hypermobility and instability. Mechanically, various tissues from EDS patients exhibit lowered elastic modulus and lowered ultimate strength. This change in mechanics has been associated with EDS symptoms. However, recent evidence points toward a possibility that the comorbidities of EDS could be also associated with reduced tissue stiffness. In this review, we focus on mast cell activation syndrome and impaired wound healing, comorbidities associated with the classical type (cEDS) and the hypermobile type (hEDS), respectively, and discuss potential mechanobiological pathways involved in the comorbidities.

## Introduction

Ehlers-Danlos Syndromes (EDSs) are a group of connective tissue disorders (Malfait et al., 2017). Currently, within the 2017 classification system, the disorders are grouped into 7 different subclasses with one more recently discovered type having yet to be named and fitted in the classification system (Malfait et al., 2017; Blackburn et al., 2018). The genes involved in every type except one have been identified (Malfait et al., 2017). Most of identified genes, if not all, are associated with components within collagen matrix, e.g., collagen I, III, V, or with assembly or crosslinking of the procollagen fibrils, e.g., procollagen N-proteinases, lysyl hydroxylase or tenascin XB, etc. Accordingly, all EDS subclasses exhibit some level of changes in collagen microarchitecture (Royce et al., 1990; Hausser and Anton-Lamprecht, 1994; Burch et al., 1997; Colige et al., 2004; Malfait et al., 2007; Fukada et al., 2008; Rohrbach et al., 2011; Hermanns-Lê et al., 2012; Kapferer-Seebacher et al., 2016; Blackburn et al., 2018; Van Damme et al., 2018; Ayoub et al., 2020; Delbaere et al., 2020; Kosho et al., 2020). Each EDS subclass is associated with stretchiness or rupture of skin and artery or hypermobility of joint (Bowen et al., 2017; Brady et al., 2017; Byers et al., 2017; Tinkle et al., 2017; Blackburn et al., 2018). Despite identified genetic defects, molecular explanation of how the genetic defects lead to clinical phenotypes that are assessed for EDS subclass determination has been unclear (Malek and Köster, 2021). Since defected genes are related to collagen formation, it has been assumed that microarchitectural changes due to those genetic defects can give rise to clinical abnormality exhibited in the EDS patients (Kobayasi, 2004). However, recent evidence shows that the structure alone is not a sole factor that determines clinical severity (Proske et al., 2006; Hermanns-Lê et al., 2012; Angwin et al., 2019). As an alternative, mechanobiological factors appear to be a key player that induces the EDS symptoms. For example, changes in adhesion and cytoskeletal organization, key mediators for mechanotransduction, are observed, along with defects in migration and contractility, in dermal fibroblasts from cEDS, hEDS and vEDS (Viglio et al., 2008; Zoppi et al., 2018). How EDS affects fibroblast dysfunction has been well discussed in a recent review article (Malek and Köster, 2021). However, how extracellular changes in EDS, particularly in the two most common types, hypermobile type (hEDS) and classical type (cEDS), can promote comorbidities of EDS have just begun to be revealed. In this review, we discuss how aberrant collagen mechanics can lead to mast cell activation syndrome and impaired wound healing, comorbidities found in hEDS and cEDS, respectively. The review begins with introducing findings about changes in tissue stiffness and extensibility in both EDS types. Then we summarize recent discoveries regarding potential linkages between mechanics and the two comorbidities and predict potential mechanobiological pathways involved in each comorbidity.

### Ehlers-Danlos Syndrome Pathophysiology Associated With Altered Collagen Microstructure

Out of 14 types (Malfait et al., 2017), most patients have one of the two most common types, hEDS and cEDS (Malfait et al., 2010; Tinkle et al., 2017). hEDS is characterized by hypermobile joints and joint instability along with other minor signs of reduced strength of connective tissues such as mitral valve prolapse, piezogenic papules, and soft stretchy skin (Tinkle et al., 2017). cEDS has similar symptoms to hEDS although cEDS-induced joint involvement is generally less severe whereas skin involvement is more severe than ones by hEDS. Skin in patients with cEDS is fragile, soft, and stretchy. Wounds take longer to heal and result in atrophic scaring (Bowen et al., 2017).

**FIGURE 1:**
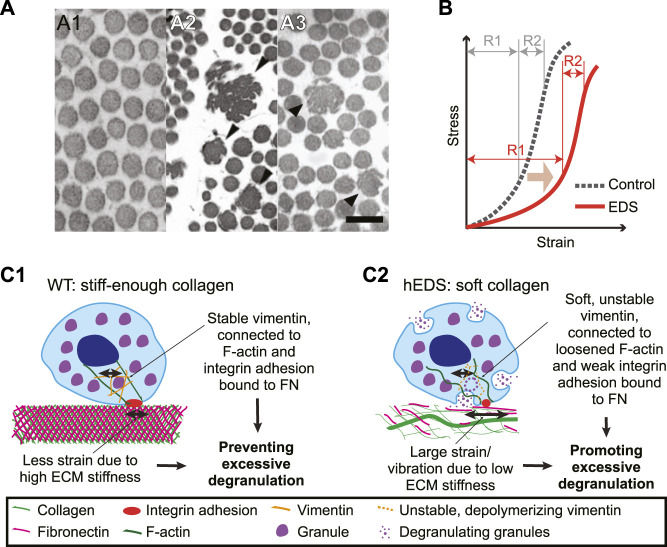
Collagen in EDS is irregular in microarchitecture, softer in mechanical behavior, and might induce mast cell degranulation. **(A)** Transmission electron microscopy images of normal skin biopsy **(A1)**, adapted from (Malfait et al., 2013), cEDS skin biopsy **(A2)**, adapted from (Angwin et al., 219), and hEDS skin biopsy (A3), adapted from (Hermanns-Lê et al., 2012). Note that collagen fibrils are enlarged (black arrowheads) and have irregular shapes in **(A2)**, and that occasional irregular fibrils depicted by black arrowheads in **(A3)**. Scale bar: 200 nm for all panels. **(B)** A typical nonlinear stress-strain curve of a skin tissue of control (dotted line) and EDS (red line). Note that EDS shifts the curve to the softer regime (light brown arrow) and that the softening is distinctly present only in the small stress regime (R1). For high stress regime (R2), there is no significant difference between control vs. EDS skin. Illustrated graph recreated by adapting (Grahame and Beighton, 1969). **(C)** Potential mechanobiological mechanism for MCAS in hEDS. **(C1)** In extracellular environment from control cases, stiff-enough collagen, intertwined with fibronectin, provides small strain or small magnitude of vibration. A mast cell adjusts its own stiffness by adjusting expression level and organization of vimentin intermediate filaments (orange), which help secure granules inside the cell. Vimentin is connected to F-actin and integrin α_V_β_3_, α_5_β_1_, and α_IIb_β_3_.*via* plectin. F-actin could also be in high tension due to the stiffness sensing via integrin adhesions. The stable vimentin and increased cell stiffness allows only small strain or vibration, which can help prevent excessive degranulation. **(C2)** In hEDS, irregular collagen organization results in overall low ECM stiffness, allowing large strain or vibration. The low ECM stiffness is sensed by integrin adhesions, which induces down-regulation of F-actin tension and cell stiffness by poor vimentin expression and polymerization. This results in poor vimentin organization around granules, unstable immobilization of granules, and ultimately promotion of excessive degranulation. Vimentin filaments were illustrated for only one part of a cell to contrast the encapsulation vs. loose organization between WT vs. hEDS, respectively.

One common component that is altered in both patients with hEDS and cEDS is collagen fibril structure. In both types, large, irregularly shaped collagen fibrils have been observed from the skin biopsy samples (Figure 1A). In addition, in patients with cEDS, overall collagen fibril diameter has been found to be larger than patients without EDS (Hausser and Anton-Lamprecht, 1994). In cEDS, the increase in diameter is caused by haploinsufficiency in collagen V. Unlike collagen I, which has a straight triple helix structure, the N-terminal end of the collagen V is globular. This structural difference results in steric hindrance limiting the diameter of collagen fibrils (Mak et al., 2016). Collagen V haploinsufficiency thus increases collagen diameter by allowing parallel collagen fibril assembly. The cause for the abnormal morphology in collagen fibrils in hEDS tissue is unknown (Tinkle et al., 2017). This altered collagen architecture appears to trigger different adhesion and cytoskeletal response by fibroblasts within connective tissue (Zoppi et al., 2018). For example, in hEDS, cEDS, and vEDS, which have different sources for altered collagen architectures, fibroblasts display similar changes in gene expression for integrin heterodimer expressions (Chiarelli et al., 2019b). This finding suggests that regardless of underlying cause of abnormal ECM architecture, the altered ECM architecture itself can induce fibroblasts dysfunction. One important parameter controlled by the microarchitecture is the mechanical stiffness, which we will focus in this review.

### Soft Tissue Mechanical Properties

Thick fibers in collagen gel have been putatively associated with high local stiffness. Collagen gels *in vitro* with thicker fibrils has been measured stiffer than collagen consisting of thin fibers, which has also been computationally modeled *via* fiber-based mechanics model (Seo et al., 2020). In cEDS tissue, however, this positive correlation has not been observed. Rather, the cEDS connective tissue, where collagen fibrils are larger than normal, has been reported softer than normal (Hausser and Anton-Lamprecht, 1994; Nielsen et al., 2014). A rheological measurement, done by measuring skin deformation curve over time in response to a sudden constant vacuum force followed by relaxation, has also shown that cEDS tissue is consistently softer than normal skin (Catala-Pétavy et al., 2009). This finding implies that the fiber diameter alone cannot predict the bulk mechanical property when it co-resides with other cells and ECM components as in the skin. The findings also suggests that reconstituted collagen gel might not represent the mechanical properties of collagen in the skin because it lacks the bundling of fibrils into fibers and the anisotropic orientation of collagen fibrils (Piérard and Lapière, 1987; Salvatore et al., 2021). Moreover, as seen in Figure 1A2, not all of collagen fibrils in skin appear to be larger in diameter in cEDS, but there are significant number of thinner fibers as well. Yet further systematic study is needed to elucidate the functional relationship between heterogeneous collagen organization and gel stiffness.

In hEDS, it is likely that the skin tissue is soft and hyperextensible, but not as much as in cEDS. When measured using a soft tissue stiffness meter, which measures the percentage change in distance between two dots drawn on the back of the hand when the skin is stretched (Farmer et al., 2010), a significant increase in extensibility has been observed in the hEDS group compared to control group (Remvig et al., 2009). However, depending on the test methods or the sample size, such significant extensibility has not been always guaranteed. When a suction cup method (Piérard et al., 2013) was used, for example, the increase in extensibility became statistically insignificant (Remvig et al., 2009). Using the same method, the reduction in the elasticity has been observed only in cEDS but not in hEDS (Catala-Pétavy et al., 2009). It is worth noting that small sample sizes were used in both studies, e.g., with 6 and 18 people, respectively, in the hEDS groups, which may not have provided sufficient statistical power. For hEDS, the connective tissue in other tissues than in the skin appears to be more distinctly and consistently softer than control group. When non-invasively measured using dynamometer during isometric plantar flexion, reduction in Achilles tendon stiffness has been observed from hEDS patients, along with larger maximal joint angle (Rombaut et al., 2012). Similarly, when the strain elastography, which estimates tissue elasticity by measuring perpendicular deformation in response to small strain induced by ultrasound, was used, the relative stiffness, represented by strain ratio and strain index, has been found to be lower in the brachioradialis muscle, patellar tendon, and Achilles tendons of patients with hypermobile disorder (Alsiri et al., 2019).

Importantly, as in typical animal soft tissue, the human skin tissue exhibits a nonlinear mechanical behavior (Figure 1B, dotted line) (Fung, 1993; Xu et al., 2008; Annaidh et al., 2012). Strain-stiffening behavior has been associated with continuous alignment of collagen with stretch (Bancelin et al., 2015). EDS, especially classical type, lowers and widens the stress-strain curve in low stress regime (Figure 1B, red line), likely due to altered microstructural change in collagen organization (Bancelin et al., 2015). Accordingly, depending on the strain regime where the skin-extending test is performed, the acquired tissue elasticity can be widely variable. When the second strain regime was used for the elastic modulus estimation, the estimated stiffness displayed no statistical difference between EDS specimen and control ones (Grahame and Beighton, 1969). In contrast, the EDS tissue’s elastic modulus is significantly reduced when it is estimated from the first, i.e., small, strain regime (Grahame and Beighton, 1969).

### Mast Cell Activation Syndrome and Mechanosensitivity of Mast Cells Within Hypermobile Type Ehlers-Danlos Syndromes

Mast cell activation disorder (MCAD) is a family of immunological disorders in which mast cells degranulate, i.e., release their granules containing histamine and other substances, unusually easily (Hamilton, 2018). Mast cells are derived from multipotential hematopoietic stem cells (MHSCs) which then differentiate into mast cell progenitors (MCPs). These MCPs then leave the circulatory system and migrate into connective and mucosal tissue where they proliferate and differentiate into mast cells (Kitamura et al., 2007). Mast cells are filled with granules filled histamine, heparin, and various cytokines among other pro inflammatory molecules. When triggered, mast cells degranulate, i.e., release the contents of their granules into the surrounding tissues (Krystel-Whittemore et al., 2016). Accordingly, too early and/or too much degranulation, which is the case of MCAD, can cause allergic reactions. Typical symptoms of these reactions are pain, fatigue, itching, flushing, dizziness, abdominal cramps, and diarrhea (Jennings et al., 2018).

Mast cell activation syndrome (MCAS) is a subtype of MCAD characterized by mast cell activation due to abnormal sensitivity of mast cells without being associated with mast cell proliferation. Around 25% percent of patients with hEDS have MCAS (Mcgillis et al., 2020). However, the MCAS-hEDS association has not been robustly supported with clinical evidence partly due to changing criteria of hEDS classification (Kohn and Chang, 2020). MCAS causes allergic symptoms when a patient is exposed to ‘a trigger’ (Hamilton, 2018). The antigen within the trigger promotes immunoglobulin E production. Binding of antigen to IgE triggers degranulation process (Krystel-Whittemore et al., 2016). The trigger includes matters that cause classical allergic responses such as food. However, it also includes heat and mechanical stimuli.

One mechanical stimulus is the physical vibration (Jennings et al., 2018). hEDS, by being easily deformable, likely increases probability to allow higher magnitude of vibration that triggers degranulation. A mediator for this vibration-induced degranulation is a transmembrane protein adhesion G protein-coupled receptor E2 (ADGRE2). ADGRE2 consists of an extracellular α subunit and a transmembrane β subunit. The protein is initially expressed as one unit but undergoes posttranslational modification by cleaving itself into an α subunit and a β subunit, held together by non-covalent bonds. While the α subunit is attached to the β subunit, it remains in its inactive state. Vibration can activate ADGRE2 by breaking its α subunit away from β subunit, which triggers degranulation (Olivera et al., 2018). Whether ADGRE2 is overexpressed in hEDS, or whether the magnitude of the vibration is a determinant of ADGRE2’s conformational change is yet to be determined. An *ex vivo* study supports the idea in the latter by showing that the magnitude of mechanical strain is a determinant of degranulation. When fibrotic rat lungs were ventilated at either low, control pressure of 5 cmH_2_O or high pressure of 30 cmH_2_O, more mast cell degranulation was observed in the high pressure-based ventilation group than in a lower pressure condition. This demonstrates that strain-induced degranulation is also strain magnitude-dependent (Shimbori et al., 2019).

Another component that may endow degranulation process with mechano-sensitivity is an intermediate filament vimentin. Intermediate filaments play a role in determining cell stiffness by resisting compression (Brown et al., 2001; Ingber, 2003). In mast cells, vimentin encapsulates the granules and immobilize them within the cytoplasm (Dráber et al., 2012). Upon degranulation, vimentin filaments rapidly depolymerize, which facilitates exocytosis of the granule’s contents (Dráber et al., 2012). Mast cells in a vimentin-deficient mouse model have shown easier degranulation than the control (Dráber et al., 2012). Cells adapt their own stiffness to the stiffness of their underlying substrate via remodeling of the cytoskeleton (Yeung et al., 2005; Solon et al., 2007). Although not explicitly reported, it is possible that mast cell stiffness could be lowered due to reduced elasticity of the collagen and the ECM, which could elicit remodeling of the vimentin filaments. Vimentin expression is correlated with substrate stiffness (Murray et al., 2014). This makes vimentin a protein of particular interest in the investigation of the pathophysiology behind in the link between hEDS and MCAS.

To be mechanosensitive, cells need to anchor themselves to the ECM. Mast cells anchor themselves to fibronectin in the ECM using integrins α_V_β_3_, α_5_β_1_, and α_IIb_β_3_ that bind to the RGD motif (Fowlkes et al., 2013). When treated with echistatin, an inhibitor for α_V_β_3_, α_5_β_1_, and α_IIb_β_3_ by competing with RGD sequence for integrin binding, mast cells have shown reduction in degranulation in a dose-dependent manner: cells treated with the highest dose of echistatin has shown the least degranulation, comparable to the static control (Fowlkes et al., 2013). This evidence suggests that the RGD-binding family of integrins are mediators for strain-induced degranulation in mast cells. As these RGD-binding integrins do not bind collagen (Humphries et al., 2006), it is possible that the “softness” of the collagen is sensed by mast cells through cells’ binding to fibronectin and fibronectin-collagen binding. It is worth being reminded of the mechanics that the resultant stiffness of the two materials connected in series is lower than that of the softer material.

These pieces of evidence point toward a potential mechanobiological interpretation about how mast cells in hEDS tissue might be easier to degranulate. As illustrated in Figure 1C, wild-type (WT) ECM is relatively stiff enough owing to well-organized collagen network. WT mast cells bind the ECM *via* fibronectin-binding integrins. Potential strain or vibration, in response to the cell-generated force or external forces, is relatively small due to high ECM stiffness. Mast cells adapt to the high ECM stiffness by upregulating F-actin tension and vimentin expression and stiffness. The stable and stiff-enough vimentin protect mast cells from excessive degranulation (Figure 1C1). In hEDS, however, a large strain or vibration magnitude is possible due to irregular collagen network and resulting softness in the ECM. Low stiffness might disable the contractility in F-actin and expression and organization of vimentin, which ultimately facilitate the cytoplasm mechanically unstable. The larger strain and unstable vimentin potentially help promote excessive degranulation (Figure 1C2). Another possibility is that the integrin adhesions can be a direct input for IgE’s activation because a study with rat basophilic leukemia mast cells has shown the adhesion protein (e.g., talin, vinculin and paxillin)-IgE colocalization and connection to F-actin (Torres et al., 2008). Softened ECM can thus induce a more direct effect on IgE via weak integrin adhesions forming in hEDS.

### Wound Healing in Classical Type Ehlers-Danlos Syndromes

One of the more challenging dermatological issues caused by EDS to treat is impaired wound management. Because of the fragility, the skin of cEDS patients splits open easily (Malfait et al., 2010). Surgical repair of these wounds is complicated by the fragility of skin in cEDS, e.g., stitches ripping through the skin. Once occurred, wounds in cEDS tend to take significantly longer to heal than wounds in control patients (Bowen et al., 2017). Furthermore, even after closure, widening of the scars tends to occur if tension is placed on them. Scars in cEDS are frequently widened and atrophic (Bowen et al., 2017).

Collagen V mutations present in cEDS have been recently associated with downregulation (e.g., SPP1, EDIL3, and PAPPA) or upregulation (e.g., IGFBP2 and C3) of genes in fibroblasts, which encode soluble and matricellular proteins involved in wound healing *in vivo* (Chiarelli et al., 2019a). Reduced migration and impaired wound healing have been observed from *in vitro* fibroblasts as well (Viglio et al., 2008). Chiarelli and colleagues have found that the changes in gene expressions can be replicated by mutated collagen V expression (Chiarelli et al., 2019a). Pathways from collagen V mutation to gene expression for wound healing, however, have remained to be investigated.

A factor that might play a critical role in the impaired wound healing in cEDS is the ECM stiffness that is significantly lowered in the case of cEDS due to lack of collagen V. The change in stiffness can affect the wound healing in many ways. First, fibroblasts’ differentiation to myofibroblasts, which plays an important role in wound healing, is regulated by the ECM stiffness (Huang et al., 2012). During wound healing, fibroblasts migrate from the edges of the wound and differentiate into myofibroblasts. Myofibroblasts contract the edges of the wounds, lay down the ECM and compact the ECM by exerting high contractile force (Darby et al., 2014). Similar to mesenchymal stem cell differentiation into bone lineage (Engler et al., 2006), fibroblasts are differentiated into myofibroblasts at a higher degree in stiff ECM than in soft substrates (Walraven and Hinz, 2018; Seo et al., 2020). Thus, we speculate that the reduced ECM stiffness in cEDS impairs myofibroblast differentiation, which again diminishes the overall wound healing process. This possibility needs further controlled experiments to evaluate.

Second, wound healing requires fibroblasts to actively migrate into the wound site. Although fibroblasts migration is directed by chemical cues such as cytokines and growth factors, it is also well established that it is strongly regulated by mechanical properties, e.g., stiffness, of the ECM. Most cell types including fibroblasts display durotaxis, that is the tendency to migrate in the direction of a stiffer surface (Lo et al., 2000; Sunyer et al., 2016). There are currently two main models for explaining durotaxis: the random walk model and the molecular clutch model. In the random walk model, the cell creates protrusions in all directions. Since the adhesions that form to stiffer substrates are more persistent than those formed on softer substrates, the net movement of the cell is in the direction of the stiffer substrate (Novikova et al., 2017). In the molecular clutch model, on stiff substrates, there is frictional slippage between flowing actin and adhesion protein (e.g., talin) whereas the interaction between actin and adhesion periodically builds up load on softer substrates until the strain reaches their failure point at which point it fails catastrophically allowing the substrate to snap back to its original position. The load-and-fail dynamics promote faster retrograde flow of actin than the frictional slippage, resulting in retraction of the cell on softer substrates and protrusion on stiffer thereby causing the cell to move in the direction of the stiffer substrate (Chan and Odde, 2008). Therefore, the lowered ECM stiffness observed in cEDS might impair wound healing by slowing down fibroblast migration.

Phenotypically, collagen V allows for cell adhesion to fibroblasts (Yamamoto et al., 1992; Kihara et al., 2004), and promotes fibroblast contractility (Berendsen et al., 2006), implying that absence of collagen V synthesis in cEDS downregulates fibroblast adhesion and contractility. Fibroblasts from a cEDS-exhibiting mouse, thus unable to synthesize collagen V, have shown reduction in migration and proliferation as well (Denigris et al., 2016). Similarly, cells derived from skin biopsies from patients with cEDS display significant reduction in expression of α_2_β_1_ and α_5_β_1_ integrins, major receptors for collagen types I-VI and fibronectin, respectively, via less-organized collagen network (Zoppi et al., 2004). Despite this reduction, cells can survive by increased expression of α_V_β_3_ integrins and avoiding anoikis, a type of apoptosis that is the result of an adherent cell becoming detached from its substrate (Zoppi et al., 2004). This has been associated with pathways independent of focal adhesion kinase (FAK). Typically, in adherent cells, FAK signaling prevent anoikis by inhibiting p53 activity which is responsible for cell cycle arrest and apoptosis (Lim et al., 2008). FAK is recruited to integrin adhesions after integrins’ binding to the ECM, is activated by integrin and undergoes autophosphorylation (Frisch et al., 1996; Zhan et al., 2004), and initiates a signaling cascade preventing anoikis (Hungerford et al., 1996; Gilmore, 2005; Zouq et al., 2009). However, FAK is not associated with the most common integrin α_V_β_3_ (Zoppi et al., 2008; Kuonen et al., 2018) but with α_5_β_1_ integrin that is expressed in wild-type fibroblasts (Zoppi et al., 2004). Instead, α_V_β_3_ acts together with endothelial growth factor receptor (EGFR) to trigger a separate pathway that involves the phosphorylation of paxillin, paxillin’s pp60src binding, which ultimately helps prevent anoikis (Zoppi et al., 2008).

**FIGURE 2:**
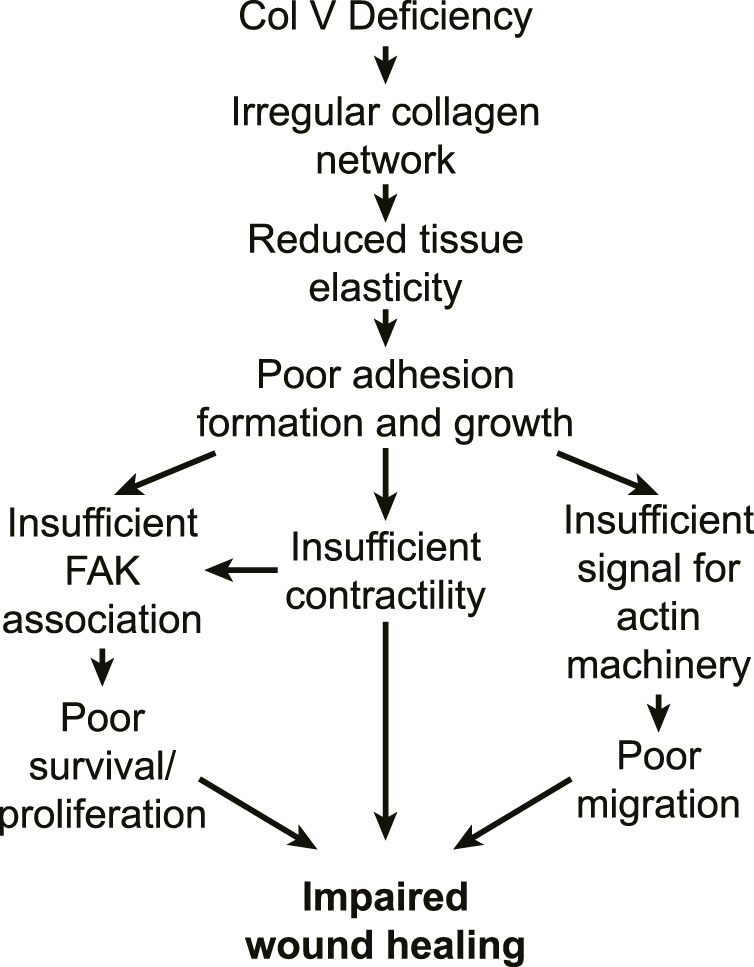
Potential pathways for impaired wound healing in cEDS tissue. In cEDS, collagen V deficiency results in irregular collagen microarchitecture, which features reduced tissue mechanics. Fibroblasts respond to the low ECM stiffness by exhibiting poor integrin adhesion formation and growth, which is unable to accommodate FAK binding. Signals from weak adhesion downregulate myosin contractility and actin polymerization, which again downregulates survival, proliferation and migration, which all impacts impaired wound healing.

The impaired cell adhesion and proliferation in cEDS as well as downregulated expressions of actin and focal adhesions can be output from mechanosensing of the ECM architecture abnormally organized by mutated/lacked collagen V. One key evidence supports this idea by exogenously expressing collagen V and showing the cEDS cells’ phenotypic change. For example, cEDS cells are restored to the wildtype phenotype with normal integrin expression and fibronectin secretion by providing an exogenous source of collagen V (Zoppi et al., 2004). Also, the cEDS-like phenotype, e.g., with the altered integrin expression, and impaired fibronectin expression, is induced in wildtype fibroblasts when their collagen V expression is blocked by collagen V-blocking antibody (Zoppi et al., 2004). Altogether, the impaired wound healing in cEDS tissue can be still attributed to softened, irregular ECM structure by lack of collagen V’s contribution, which induces poor cell-ECM adhesion formation and growth, which again transduce insufficient mechanotransduction signals that supports cell migration, survival, and proliferation (Figure 2). Detailed studies on how collagen V changes microarchitecture of the collagen network and overall ECM would shed light on further insight to the cEDS’s main comorbidity.

## Conclusion

We have reviewed the two most popular types of Ehlers-Danlos syndrome, hEDS and cEDS in a perspective of mechanobiology. We attempted to link the common mechanical feature in both hEDS and cEDS, e.g., connective tissue being softer to comorbidities associated with hEDS and cEDS: MCAS and impaired wound healing, respectively. For MCAS, we identify ADGRE2, vimentin, and the RGD binding family of integrins α_V_β_3_, α_5_β_1_, and α_IIb_β_3_ as mechanosensitive proteins that are altered in hEDS. For impaired wound healing, the reduced ECM stiffness, due to lack of collagen V, may be the main contributor to impaired myofibroblast differentiation, altered integrin expression and fibronectin secretion in cEDS. Beyond the current focus in the EDS field on uncovering the cause of hEDS and better defining what MCAS is, our review suggests that mechanobiological research using hEDS mast cells or cEDS fibroblasts would advance mechanistic understanding of phenotypic changes in the two comorbidities.
